# Unhealthy Fat in Street and Snack Foods in Low-Socioeconomic Settings in India: A Case Study of the Food Environments of Rural Villages and an Urban Slum

**DOI:** 10.1016/j.jneb.2015.11.006

**Published:** 2016-04

**Authors:** Vidhu Gupta, Shauna M. Downs, Suparna Ghosh-Jerath, Karen Lock, Archna Singh

**Affiliations:** 1 Indian Institute for Public Health–Delhi, Public Health Foundation of India, Haryana, India; 2 Menzies Centre for Health Policy, University of Sydney, Sydney, Australia; 3 London School of Hygiene and Tropical Medicine and Leverhulme Centre for Integrative Research on Agriculture and Health, London, United Kingdom; 4 Indian Institute for Public Health–Delhi, Public Health Foundation of India and All India Institute of Medical Sciences, Delhi, India

**Keywords:** trans fatty acids, fatty acids, food environment, snacks, low- or middle-income country

## Abstract

**Objective:**

To describe the food environment in rural villages and an urban slum setting in India with reference to commercially available unbranded packaged snacks and street foods sold by vendors, and to analyze the type and quantity of fat in these foods.

**Design:**

Cross-sectional.

**Setting:**

Two low-income villages in Haryana and an urban slum in Delhi.

**Participants:**

Street vendors (n = 44) were surveyed and the nutritional content of snacks (n = 49) sold by vendors was analyzed.

**Main Outcome Measures:**

Vendors' awareness and perception of fats and oils, as well as the type of snacks sold, along with the content and quality of fat present in the snacks.

**Analysis:**

Descriptive statistics of vendor survey and gas chromatography to measure fatty acid content in snacks.

**Results:**

A variety of snacks were sold, including those in unlabeled transparent packages and open glass jars. Mean fat content in snacks was 28.8 g per 100-g serving in rural settings and 29.6 g per 100-g serving in urban settings. Sampled oils contained high levels of saturated fats (25% to 69% total fatty acids) and trans fats (0.1% to 30% of total fatty acids).

**Conclusions and Implications:**

Interventions need to target the manufacturers of oils and fats used in freshly prepared products to improve the quality of foods available in the food environment of low-socioeconomic groups in India.

## Introduction

What people eat depends not only on individual and cultural factors but also on their surrounding food environment.^1^ The food environment is one of the major domains in which policies can intervene to improve the availability, affordability, and acceptability of healthier food.^2^ By improving nutrition labeling, offering healthier foods, setting standards in public institutions, using economic tools to address food affordability, restricting food advertising, improving the quality of the food supply, and setting incentives and rules to create a healthy retail environment, the food environment can better support consumers to make healthier food choices.^2^

Most research on food environments has been conducted in the US and other high-income countries.^1^ In the US, studies have shown that lower-socioeconomic communities tend to be characterized by the high availability of convenience and energy-dense foods of little nutritional value and the low availability of fresh produce and other nutritious foods.^3^, ^4^ Barriers to access can make it difficult for people to choose healthy food.^5^, ^6^, ^7^ However, there is a paucity of research assessing the food environments of low-socioeconomic status (SES) settings in low-income (defined as those with a gross national income per capita of ≤ $1,045 in 2013) and middle-income countries (defined as those with a gross national income per capita of > $1,045 but < $12,746) (LMICs). Therefore, it is important to examine the situation in LMICs, because little is known about the nature of their food environments.

Many LMICs worldwide are currently undergoing a nutrition transition: a shift from traditional dietary patterns toward a more Western diet consisting of energy-dense foods high in fat, sugar, and salt.^8^, ^9^, ^10^ The transition, which has been linked to a rise in diet-related noncommunicable diseases, is rooted in globalization.^11^ As a result, many countries now face a double burden of diet-related diseases in which undernutrition and overnutrition coexist, even within the same household (HH).^12^, ^13^, ^14^

Although the dietary intakes of lower-socioeconomic groups in LMICs are often limited by the scarcity of food, it may no longer be the main factor affecting energy intakes in some settings; instead, the availability of cheap, energy-dense foods, including those from street vendors, may result in increased energy intake.^15^ Street and snack foods have become a significant source of food for many people in LMICs as people migrate from rural to urban areas.^16^ In particular, low-income populations often depend almost exclusively on food prepared by street vendors.^16^ Unfortunately, many vendors sell food of suboptimal nutritional quality.^15^ A study conducted in India found that migrant and urban men consumed a higher proportion of energy from fat and saturated fat than do rural men, which suggests a shift toward a lower-quality diet after migration.^17^ In the absence of a diversified, nutrient-dense diet, there may be a propensity for overconsumption leading to overweight and obesity but a simultaneous failure to meet micronutrient requirements.^18^

In India, HHs in both urban and rural areas are dealing concomitantly with undernutrition and diet-related noncommunicable diseases, particularly heart disease and diabetes.^19^ Urban slum dwellers in India are often deficient in key nutrients^20^ while consuming high intakes of trans fatty acids (TFAs) from hydrogenated oils.^21^ These dietary TFAs have adverse effects on blood lipoprotein profiles and coronary heart disease risk affecting individuals and populations. The adverse effects on coronary heart disease are mediated by increases in plasma concentrations of low-density lipoprotein cholesterol and lipoprotein A, and reductions in high-density lipoprotein cholesterol, promotion of inflammation, and endothelial dysfunction.^22^ Many Indian snacks have been found to be high in TFA (≥2% of total fatty acids).^23^, ^24^ The Government of India recently took steps to limit the amount of TFA in foods by publishing a regulation setting an upper limit of 10% TFA in partially hydrogenated vegetables oils (PHVOs). This regulation was subsequently revised to a 5% limit that will come into effect in August, 2016, in addition to requiring that TFA be labeled on packaged foods.^25^, ^26^ However, it is unclear to what extent this regulation will be enforced and what substitutions vendors will make to keep consumers satisfied and maintain product demand despite the changes.

There is currently limited information about the food retail environments of low-socioeconomic groups in LMICs, particularly those living in rural areas and urban slums. Creating healthy public policies and supportive food environments can facilitate access to safe, affordable, nutritious food.^27^ To identify which policies might be the most effective in specific settings, it is important to gain a thorough understanding of the existing food environment. Thus, a project was designed that aimed to examine awareness of and use of TFAs and the feasibility of their removal from the Indian food supply chain by integrating perspectives from 3 levels: manufacturers, retailers, and consumers. The current study, which is part of the larger project, describes the retail food environment with a focus on unbranded packaged products and street foods in rural and urban low-SES settings of 2 states in North India. The objectives of this study were to (1) describe the food environment in selected low-SES rural and urban settings in North India with reference to commercially available, unbranded, packaged, ready-to-eat snacks and street foods sold by vendors; and (2) analyze the type and quantity of fat in these foods to understand the exposure to the population.

## Methods

This study analyzed the retail food environment in terms of the snacks and street foods available and sampled from the vendors in the low-SES settings in North India. The researchers determined the snack sampling strategy using data obtained from the consumer-level study. This included a survey of the dietary intake patterns with an emphasis on snacking patterns conducted in low-SES HHs (260 HH in each community). The HH surveys included a dietary intake questionnaire as well as 2 24-hour dietary recalls, which were conducted on 2 consecutive days. The dietary questionnaire, in addition to the 24-hour recalls, provided information regarding consumption of commercially prepared snacks, which was then subsequently used to determine the snack sampling strategy. Only unbranded (unlabeled, open, or freshly prepared) snacks from all vendors willing to participate were sampled (details are provided subsequently). Therefore, snacks sampled from the vendors were those consumed (if reported in the 24-hour recall) by participants in the HH survey. The authors obtained ethics approval for the study from the Public Health Foundation of India's Institutional Ethics Committees.

### Setting

The researchers examined the food environments of 3 purposively selected settings, which included 2 rural villages (Sundh and Hasanpur) in the Mewat district of Haryana and an urban slum (Chanderpuri) in the northeast district of Delhi. These communities were selected specifically to examine snacking patterns (through the HH survey) in low-SES settings in 2 adjacent states that typically consume *vanaspati* (PHVO high in TFA) and unbranded snacks made using PHVOs. Before beginning the study, the authors obtained permission to conduct the study from the local leader (a community representative such as the Councilor, *Pradhan*, or *Sarpanch*) of the study areas. The populations of the villages were 5,000 and 6,000 people, which corresponded to 525 and 800 HHs, respectively. The population of the urban slum was approximately 10,812, which represented 1,802 HHs. Data collection took place between October and November, 2012 in the villages and February to March, 2013 in the urban slum.

### Study Design

The researchers conducted a cross-sectional exploratory study that included surveying local food vendors and performing laboratory analyses of the fat content of snack and street foods sampled from the study communities. Street foods were defined as ready-to-eat foods and beverages prepared and/or sold by vendors and hawkers, especially in streets and other similar public places, and snack foods were defined as foods eaten between meals.^20^ Street and snack foods included both branded and unbranded snacks; however, the focus of this study was on unbranded snacks that were unlabeled, as well as open or freshly prepared street foods. Branded packaged snacks were defined as those manufactured by multinational companies, containing a label and brand name available in commercially available standard packages. Labels of these products included information about the net weight, nutritional composition, ingredients, brand name, standard packages, manufacturing, and expiration date. Unbranded packaged snacks were defined as those that were produced by local and district-level manufacturers but that were available in unlabeled transparent packages. Information about weight, brand, and manufacturing date was sometimes available but no nutritional composition was mentioned on the package. Open snacks included those that were not packaged and were available in jars or tins (eg, *soan papdi*, *mathri*). Freshly prepared snacks (eg, *samosas*, *kachodi* [deep-fried, salted, puffed bread made of wheat flour]) were those cooked at the time of purchase.

**Figure 1:**
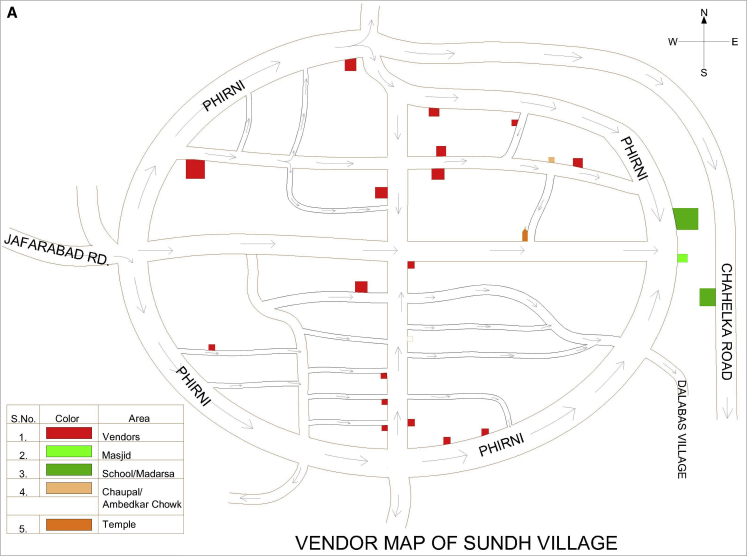

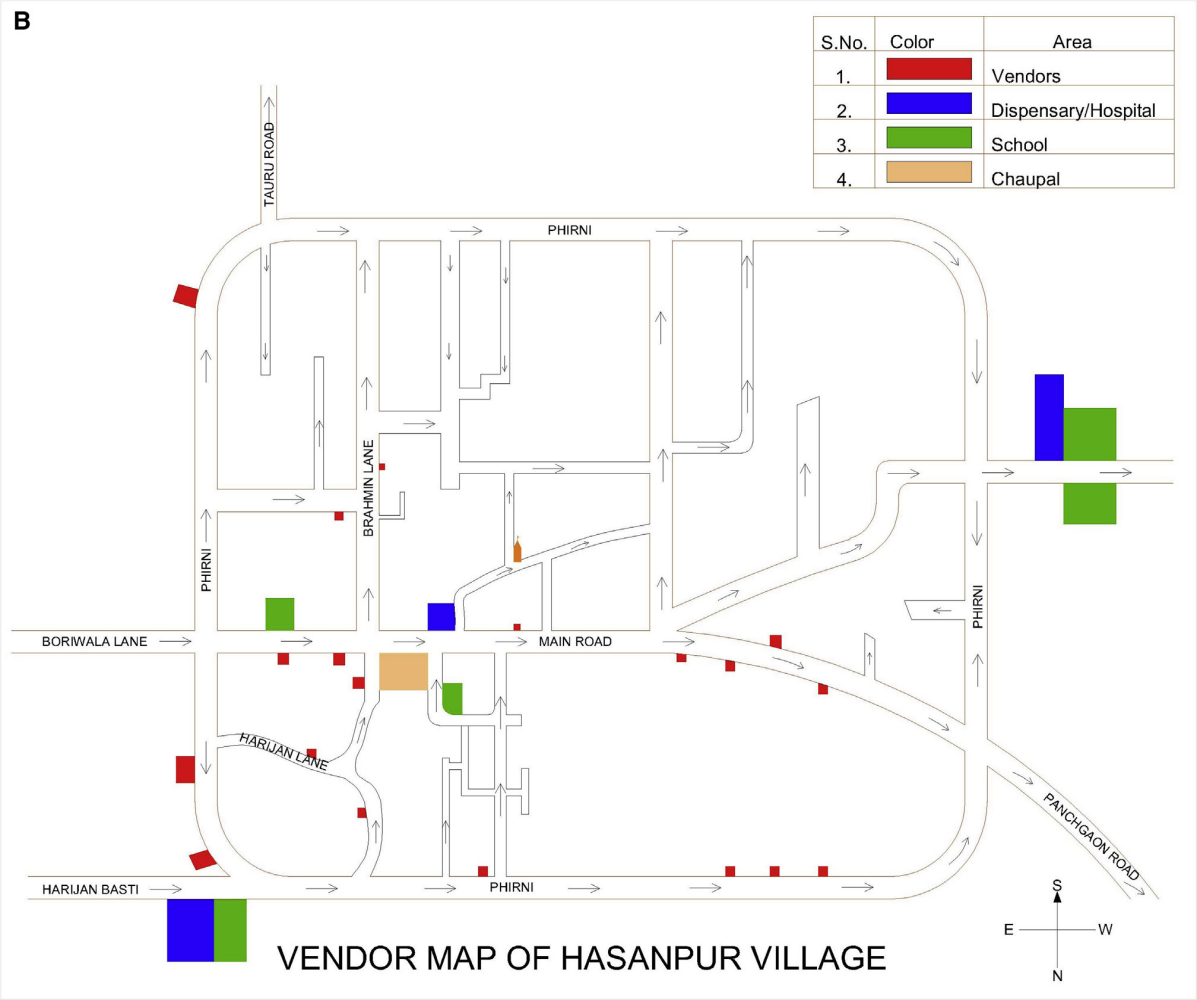
(A) Map of village (Sundh) showing the location of vendors (selling unbranded, open, and freshly prepared commercially available snack foods) and public buildings (eg, school, medical building) in the study community. (B) Map of village (Hasanpur) showing the location of vendors (selling unbranded, open, and freshly prepared commercially available snack foods) and public buildings (eg, school, medical building) in the study community.

**Figure 2:**
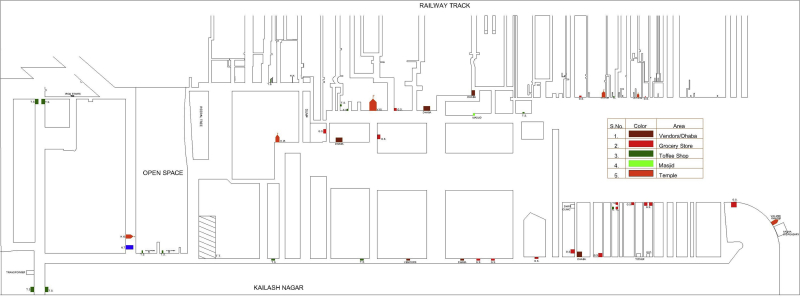
Map of urban slum (Chanderpuri) showing the location of vendors (selling unbranded, open, and freshly prepared commercially available snack foods) and public buildings (eg, school, medical building, temple) in the study community.

Before inviting vendors to participate in the study, the authors mapped the food environments (with a focus on snack foods) in the villages and the urban slum by documenting the number and location of all HHs, vendors, and public buildings (including schools, temples, etc) in each community to facilitate the survey (Figures 1 and 2). These maps were then used to describe the food environment in terms of the location of vendors with respect to the HHs, who sold unbranded, open, and freshly prepared commercially available snack foods in these survey communities.

All food vendors working in each of the communities were invited to participate in the study. All participating vendors provided informed consent and all surveys were conducted in Hindi. Responses were subsequently translated into English.

### Vendor Surveys

The research staff contacted the vendors at their shops subsequent to mapping the survey areas. The vendor survey instrument was a 17-question structured survey administered in person by the trained research staff; surveys were completed at the shops of participating vendors. The majority of the questions were open-ended (16 of 17), which allowed vendors to provide specific answers to each question. The survey questionnaire was reviewed by experts in the field (nutrition, public health, and biochemistry) before data collection; however, it was not validated. Vendor practices were explored by collecting information on the types of snack foods they sold, how they prepared them, and their use of cooking oils. Vendors were also asked about the oils they use (if they prepared snacks themselves), why they used them, where they purchased the oils, the quantity and cost of the oil, the length of time they used the oil and how they discarded it, and questions related to preferences about the oil. Moreover, vendors were asked about their awareness of TFAs and their views about the overall healthiness of oils. To assess awareness about TFAs, the researchers asked vendors if they had heard of TFAs. If they responded yes, the researchers asked them what the health implications were (both positive and negative) of TFA consumption. The average survey duration was 20–30 minutes.

### Sampling Strategy of Snacks and Oils

In addition to administering vendor surveys, the researchers obtained samples of a variety of snacks from the vendors to assess the content and fatty acid composition of the snacks. Selection criteria for the sampling of snacks included unbranded snacks that were unlabeled, open, or freshly prepared and reportedly consumed in the 24-hour dietary recall (that was conducted as part of the HH survey). In addition, the authors sampled snacks that were reported to be commonly consumed (≥3 times/wk) in the dietary questionnaire but did not happen to be consumed on the day when dietary recalls were conducted. There is a large unorganized sector of regionally popular snacks and convenience foods operating in low-SES settings. This is an established source of snacks for the local population. Therefore, snacks being sold in standard packaging (from multinational companies) and with food labels were excluded from the sampling. The types of oils and fats that were reportedly consumed in the HH survey and commonly purchased by consumers in the vendor survey were sampled from both communities. These included refined oil, mustard oil, *vanaspati*, and *desi ghee* (clarified butter). The fatty acid profile of sampled oils and fats was also analyzed.

### Fat Composition of Snacks and Oils

The snacks and oils sampled from the 2 settings were examined using gas chromatography to assess their fatty acid composition. All samples from vendors were collected at a single time point. Snack foods included unlabeled packaged products (such as biscuits and chips), open and freshly prepared ready-to-eat foods such as *samosas*, *namkeens*, and other similar items. Fat extraction and estimation was done based on Association of Official Analytical Chemists protocol 996.06 on a gas chromatograph equipped with a flame ionization detector (Nucon Series II, 5700/5765, Nucon Engineers, Delhi, India, 2005). The limit of detections was calculated based on the multiple dilution of standard fatty acid methyl ester mix. The fatty acid profiles were analyzed using a fatty acid methyl ester mix (GLC-607) and individual fatty acid esters from NuChek Prep, Inc (Waterville, Maine), to characterize and identify individual fatty acids. All analyses were done in duplicate.

### Analysis

Responses to the open-ended vendor survey questions were summarized by key themes such as type of snacks sold, vendor practices, and health awareness among vendors. All descriptive statistics for the vendor survey were conducted using Microsoft Excel (version 12.1, Washington, DC, 2008). AIMIL software (New Delhi, India) was used to edit the chromatogram (the graph of detector response against the retention time of the individual fatty acids). The chromatogram provides the range of peaks representing the specific fatty acids present in the sample. Editing is analogous to data cleaning and involves integrating the peaks eluted. Once this is completed, the individual peak areas represent the percentage contributed to the sample by individual fatty acids. Thus, the saturated, monounsaturated, polyunsaturated, and TFA content of the fats extracted from the sampled snacks was calculated. The content of each fatty acid in the sample was expressed as a percentage of the total fatty acids in that sample.

## Results

### Characteristics of Vendors

Of the 40 vendors in the villages and the 29 vendors in the slum, 27 (68% participation rate) and 17 (59% participation rate) agreed to participate, respectively. The 2 rural adjacent villages that were examined had a bus stop between them where the vendors of both villages procured the snacks for their shops; however, 7 village vendors (26%) prepared at least some of the items themselves. Six of the vendors (35%) in the urban slum prepared some of their own snacks; the remaining snacks were procured from third-party wholesalers who came to the slum to sell directly to the vendors, and from wholesale distributors and grocery shops. However, snacks sampled from vendors for testing included those purchased from third-party vendors or prepared fresh as long as they were also unbranded.

### Types of Snacks Sold by Vendors

Vendors sold both branded and unbranded packaged snacks as well as open and freshly prepared street foods. The total number of snacks available was 33 and 52 in the rural area and the urban slum, respectively. Thirty-six percent of snacks were branded packaged snacks in the rural setting compared with 33% in the urban setting. The remaining snacks (64% rural and 67% urban) were unbranded. In addition to unbranded snacks, confectionery and freshly prepared snacks (8 in the rural area and 13 in the urban slum) were available.

### Vendor Practices

A variety of oils including soybean and other unspecified refined oils, *vanaspati*, and local oil brands were used by the rural and urban vendors. Vendors in both settings reported preparing snacks using refined oil to fry snacks such as *samosa*, *kachodi*, *bhatura*, *bread pakoda*, and *golgappe*. *Vanaspati* was used as shortening in these snacks by 2 (out of 7) and 3 (out of 6) of vendors in rural and urban settings, respectively. Most of the urban vendors (4 of 6) bought the oils from a local market whereas the rural vendors (5 in 7) bought them from the nearby town. Vendors indicated that they chose these oils based on the quality, including organoleptic properties (4 of 7 in rural areas; 2 of 6 in the urban area), taste (1 of 7 in the rural settings; 5 of 6 in the urban setting), traditional use (1 of 7 in the rural areas), cost (1 of 6 in the urban slum), and customer preferences (1 of 7 in the rural settings). Most urban vendors (n = 5 of 6) indicated that they would be willing to change the type of oil used provided the taste (n = 1), cost (n = 3), and customer acceptability (n = 1) were adequate; rural vendors (n = 3 of 7) were more resistant to change. However, some vendors (n = 2 of 7) stated that they would change if the quality were maintained.

### Health Awareness Among Vendors

Awareness regarding TFAs was low among vendors. Only 1 of the village vendors and 2 of the urban slum vendors stated that they were aware of TFAs and described them as being bad for health. Overall, village vendors (n = 5) indicated that they either did not know which oils could be considered better or worse for health or that mustard oil was healthy (n = 8). The majority (n = 10) of urban vendors indicated that refined oils (including soybean, mustard, and safflower) were healthy.

### Fat Content of Street and Snack Foods

The researchers sampled and analyzed 17 snack samples from the villages and 32 from the slums. Tables 1 and 2 provide the fat and fatty acid profiles of the unbranded, open, and freshly prepared snacks from both the rural and urban settings. The fat content of snacks in their usual servings ranged from 0.1 to 32 g in villages and 0.6 to 26 g in the urban slum. The mean fat content in snacks in villages was 28.8 g per 100-g serving (SD, 17.8 g) and in the urban slum it was 29.6 g per 100-g serving (SD, 12.6 g). The fat content (per 100 g) was highest in the open snack mixture (salted tidbits of wheat and lentils: 64.8 g per 100 g) and freshly prepared *kachodi* (46.6 g per 100-g serving) in rural and urban settings, respectively. The overall average serving size of all snacks combined was larger in the urban slum compared with the rural setting (41 vs 25 g, respectively); however, there was a greater variety of snacks in the urban slum. Fat (per 100 g) and TFA levels were higher in the open and freshly prepared snacks than in the unbranded packaged snacks.

Three and 7 oils and fats were sampled from rural and urban settings, respectively. Table 3 provides the fatty acid profile of the different oils sampled from both vendors and HHs. The fat content of sampled oils, expressed as a percentage of total fatty acids, contained high levels of saturated fat (ranging from 24.7% to 69.3%) and TFA (ranging from 0.1% to 29.9%). The TFA levels exceeded 20% of total fatty acids in both the *vanaspati* samples and the sample of *desi ghee* from the village market.

## Discussion

Understanding the broad food environment is crucial in terms of informing the development of policy interventions aimed at improving the quality of the food supply. As the food environments of LMICs such as India evolve, policy interventions will need to be context-specific to improve diet quality and reduce the risk of diet-related disease most effectively. The authors found that both rural villages and an urban slum setting in India had a large number of vendors selling energy-dense snacks high in fat (including saturated fat and TFA).

### Nutrition Labels

The lack of nutrition labels on snack foods in this study is problematic and was previously noted in a study conducted in the Indian state of Kerala.^24^ The majority of confectionery products in that study contained TFA and/or high levels of saturated fat, mainly palmitic acid (from palm oil).^24^ In the current study, high levels of TFA were found in open snacks that do not contain labels. These high levels of unhealthy fat are consistent with the levels found in a recent study conducted in Uttar Pradesh, India.^28^ Although India recently published a regulation requiring TFA labeling in packaged foods, it is likely that the regulation will not apply to snack foods that are sold open or freshly prepared, which contain the highest levels of TFA in the Indian food environment.^25^, ^26^ Moreover, packaged unbranded snacks produced by the informal sector will likely evade regulation as well. Because many foods do not contain labels, consumers will be unable to identify the contents of foods they are consuming. Labeling regulation currently does not reach the large informal food processing and retail sectors in the country and new approaches are needed to target these foods, which reinforces the need to target more upstream steps in the food supply chain. Moreover, the use of nutrition labels is limited in India and knowledge about how to use them is low.^29^ In addition to intervening at the manufacturing level, alternative approaches to increase consumer demand for improved transparency and healthier snack food options are needed, which would in turn facilitate greater emphasis on these aspects by vendors and manufacturers.

### Food Sold by Vendors

Although a small number of vendors in this study prepared their own snacks for sale, the majority purchased snacks from a third-party wholesaler. This has important implications in terms of policy interventions. Interventions aimed at improving the quality of snacks sold by vendors will need to be directed at both the manufacturers of these foods and the vendors themselves in the case of those preparing fresh snacks. Vendors in this study were aware only of the type of cooking fat used in the products that they prepared themselves. Of those who prepared their own snacks, they seemed to be amenable to changes in the type of cooking fat used. The Singapore Health Promotion Board recently began an initiative called the *Healthier Hawker Program*, which aims to reduce the saturated fat content of cooking oils used by street vendors.^30^ To ensure that there was an affordable supply of healthier oils, the Health Promotion Board worked with local manufacturing companies to increase the supply of blended oils containing 25% less saturated fat.^30^ Moreover, the healthier ingredients symbol program, which is part of the *Healthier Hawker Program*, allows vendors to put up a sign indicating the use of healthier ingredients if TFA levels are < 0.5 g per 100 g of oil and saturated fat levels are < 38 g per 100 g of oil.^30^ This initiative increases vendor access to healthier oils and could also help create consumer demand for healthier oils in ready-to-eat foods.

### Oil Use by Vendors

Although many vendors (of those preparing their own snacks) used refined oils to fry freshly prepared snacks, and identified refined oils as being healthy, many still used *vanaspati* containing high amounts of TFA as the shortening in preparing items such as *samosa*, *kachodi*, and other similar snack items. In addition to the high levels of TFA in some snack foods, there were signs of possible adulteration of the oils used by vendors. For example, levels of saturated fat were higher than would be anticipated in pure mustard oil, as were TFA levels in the *desi ghee* available in rural markets. This reinforces the need to go beyond nutrition labeling policies to address fat quality in the foods sold and prepared by street vendors. Reducing the upper limit of allowable TFA in PHVOs and ensuring that it is adequately enforced would ensure that foods sold and prepared by vendors do not contain TFA, which would reduce the risk of cardiovascular disease for HHs that frequently consume these foods.^31^

The majority of vendors were not aware of TFA or the health risks associated with its consumption. It is likely that increased awareness and capacity for reformulation of recipes among vendors will be needed to ensure a shift from the use of *vanaspati* to healthier oils in some traditional snacks. Programs aimed at improving street vendor practices tend to focus on food safety and hygiene issues; however, other practices such as the use of oil could be targeted in these programs. In Ghana, food vendor knowledge increased and practices improved after the vendors underwent training on food safety and hygiene practices.^32^ The Food Safety and Standards Authority of India has already committed to improving street vendor practices. By moving beyond the narrow definition of food safety, the Authority could facilitate broader improvements in street vendor practices with the potential to improve the quality of foods that are available in low-income settings.

### Nutrition Education

Nutrition education has an important role in the selection of oils and fats for preparing snacks. This is not restricted to vendors preparing the snacks or manufacturers producing the cooking oil; it extends to the consumers who actually consume these fats and oils in the form of cooking oil or as snacks purchased in the market. Nutrition education is needed at every level of the food supply chain, from manufacturers to consumers, to be an effective approach to decreasing the availability of TFA in the food supply and its consumption at the consumer level. Manufacturers and vendors need to be informed of the benefits of using blended oils to decrease saturated fat and TFA content. In Singapore, blending of oils by manufacturers led to a reduction in saturated fat levels in these oils without jeopardizing taste.^30^ Through negotiations with suppliers, the cost of these healthier oils remained low, which made it a feasible oil option for vendors.^30^ Consumers need to be encouraged to select and use healthier oil for cooking and deep-frying purposes. At the same time, alternate ways of cooking traditional snacks, such as baking rather than frying, should be encouraged.

A limitation of this study was the relatively small sample size. Thus, findings of the current study cannot be generalized to broader food environments across India. Only 60% of vendors participated in the current study and few vendors among these actually prepared fresh snacks compared with vendors who sold ready-to-eat snacks procured from third-party wholesalers. Therefore, the authors observed limited knowledge among the vendors about the type of oils used to prepare the products. The survey instrument had not been previously validated. Finally, the authors did not investigate differences between the vendors who participated in the survey and those who did not, which could have influenced the study findings. However, the authors did not observe obvious differences between the 2 groups.

## Implications for Research and Practice

The food environments in low-SES communities in India are saturated with energy-dense street and snack foods containing high amounts of TFA and saturated fat. Because it is unlikely that consumers will stop purchasing these snacks in the short term, it is vital to improve the quality and transparency of the contents of snack foods that are available. Interventions aimed at targeting the manufacturers of oils and fats used in freshly prepared products, wholesale producers of unbranded snacks, and vendors preparing their own snacks have the potential to improve the quality of foods available in the food environment of low-SES groups in rural and urban areas in India. However, this will require research and the development of products that are cost-effective for manufacturers and vendors and also have a healthier fatty acid profile.

Incentives upstream in the food supply chain are needed to ensure adequate and cost-effective technology transfer to manufacturers for the production of healthier alternatives to oils high in unhealthy fats. Both government and nongovernmental organizations in India should consider adopting programs such as the *Healthier Hawker Program* (Singapore) that try to encourage vendors to swap to healthier oils. This will need to be coupled with initiatives aimed at improving both vendor and consumer awareness related to TFA. One way to accomplish this could be to implement a social marketing campaign that provides information about the use of healthier fats and oils for cooking traditional Indian snack recipes. Moreover, information about the importance of consuming healthier fats and oils should be included in the Indian Dietary Guidelines,^33^ which currently focus primarily on combating undernutrition. The guidelines should emphasize the use of indigenous vegetable oils high in polyunsaturated fat rather than those high in saturated fat and TFA. Without improved awareness, it is unlikely that consumers will demand healthier snacks. Because there may be a preference for lower-cost oils that are often high in saturated fat and/or TFA,^34^ blended oils with different fatty acid profiles could be used to produce healthier oil available at a competitive price. The technology exists to make a product that is TFA-free with moderate amounts of saturated fat^35^, ^36^ and it is currently being used in Europe and North America, but ways are needed to make it a cost-effective solution in India and other low- and middle-income settings.

## Figures and Tables

**Table 1:** Total and Trans Fat Content of Snacks Sampled From Village Settings

Snack Name and Description	Weight of Snack, g^a^	Total Fat, g	Fat per 100-g Serving, g	Saturated Fatty Acids^b^	Monounsaturated Fatty Acids^b^	Polyunsaturated Fatty Acids^b^	Trans Fatty Acids^b^
Packaged unlabeled snacks							
*Bhindi* (wheat puffs)	4.00	0.32	8.00	61.27	19.11	18.45	0.53
Sweet biscuits (1 biscuit)	5.40	0.76	14.07	53.72	33.28	11.43	0.00
Salted biscuits (1 biscuit)^c^	4.50	1.56	34.67				
*Rusk* (dry biscuit, twice-baked bread)	8.00	1.68	21.00	44.42	13.70	40.77	0.29
*Pao* (bread buns)	2.00	0.08	4.00	34.13	18.96	41.53	1.15
*Fan* (wafer-shaped, flaky, salted pastry puffs)	28.78	6.91	24.01	87.00	2.17	0.51	10.25
Open snacks							
Mixture (salted tidbits in a pack-mix of wheat and lentils)	10.00	6.48	64.80	42.79	36.71	12.22	0.39
*Mathri* (fried, salted thick wheat crackers)	11.00	6.29	57.18	18.19	22.79	57.76	1.26
*Moong dal* (savory lentils)	21.00	3.74	17.81	59.39	19.41	18.15	0.60
*Soan papdi* (a flaky sweet made of graham flour)	20.00	7.80	39.00	62.94	12.05	15.03	9.86
Freshly prepared snacks							
*Samosa*^a^ (potato-stuffed, deep-fried, salted, refined-wheat flour pockets)	79.25	23.46	29.62	60.21	14.25	13.27	10.35
*Kachori* (deep-fried, salted, puffed bread made of wheat flour)	55.70	17.82	31.99	45.73	1.84	31.49	11.67
*Bread pakora* (deep-fried graham flour–coated bread triangles)	67.50	32.13	47.60	25.95	23.24	41.36	0.37
*Bhatura* (deep-fried, leavened, puffed bread made of refined wheat flour)	33.00	3.30	10.00	47.01	37.40	9.09	0.00

a The weight reflects the serving size in which the snack was sold in the village markets, with the exception of biscuits, which refers to the weight of 1 biscuit.

b Expressed as a percentage of total fatty acids.

c Fatty acid profile not available.

**Table 2:** Total and Trans Fat Content of Snacks Sampled From Urban Slum

Snack Name and Description	Weight of Snack, g^a^	Total Fat, g	Fat per 100-g Serving, g	Saturated Fatty Acids^b^	Monounsaturated Fatty Acids^b^	Polyunsaturated Fatty Acids^b^	Trans Fatty Acids^b^
Packaged unlabeled snacks							
*Chakri* (deep-fried spiral wheat crackers)	21.42	7.88	36.79	63.94	16.79	18.73	0.97
*Kachri* (rice puffs)	9.06	3.22	35.54	48.48	38.90	10.61	0.33
*Fun Pop* (rice puffs, chips)	3.89	0.61	15.68	54.02	27.01	18.46	0.42
*Jo's Onion Rings* (chips)	14.65	6.62	45.19	57.22	26.82	14.91	0.45
Bun (2 pieces)	78.20	3.28	4.19	39.22	5.35	49.94	4.56
*Rusk* (dry biscuit, twice-baked bread)	23.94	3.16	13.20	44.32	22.76	40.76	0.53
Open snacks							
*Mathri* (fried, salted thick wheat crackers)	21.35	8.22	38.50	87.34	5.05	2.62	4.43
Biscuits, unpackaged (4 biscuits)	20.26	8.27	40.82	88.45	9.00	0.08	0.35
*Bhujia* (fried, salted snack made of graham flour and shredded)	31.96	12.66	39.61	59.62	22.71	16.43	0.46
*Moong dal* (savory lentils)	18.29	5.01	27.39	33.28	8.94	55.27	1.26
*Besan ladoo* (a ball-shaped sweet made of fried graham flour)	33.33	7.93	23.79	62.45	29.17	7.42	0.37
*Besan barfi* (sweet graham flour squares)	49.10	10.51	21.41	68.37	5.40	0.50	22.96
*Maida barfi* (sweet flour and condensed milk triangles)	44.52	2.58	5.80	50.77	26.38	20.19	0.00
*Soan papdi* (a flaky sweet made of graham flour)	49.01	9.90	20.20	49.81	38.54	11.14	0.29
*Besan papdi*^c^ (a flaky sweet made with graham flour) (1 piece)	32.80	16.53	50.40				
*Besan papdi*^c^ (a flaky sweet made of graham flour with ground nuts) (1 piece)	30.13	17.47	57.98				
Confectionery snacks							
Cream roll	58.00	18.44	31.79	86.79	2.10	0.60	2.88
Pastry	32.53	6.90	21.21	67.50	16.89	14.76	0.26
Patty	89.34	26.09	29.20	69.62	6.86	11.21	10.25
*Fan* (wafer-shaped, flaky, salted pastry puffs)	23.67	7.77	32.83	90.74	2.66	0.54	4.98
Muffin	21.87	8.40	38.41	61.70	27.83	9.13	0.37
Freshly prepared snacks							
*Bhatura* (deep-fried, leavened, puffed bread made of refined wheat flour)	52.04	10.20	19.60	58.81	28.55	11.42	0.44
*Kachodi* (deep-fried, salted, puffed bread made of wheat flour)	53.83	25.09	46.61	85.40	1.81	0.31	11.77
*Bread pakora* (deep-fried graham flour–coated bread triangles)	90.10	23.79	26.40	64.07	19.93	14.59	1.06
*Namakpara* (fried, salted wheat flour cubes)	64.12	22.44	35.00	84.49	1.71	0.42	12.81
*Samosa* (potato-stuffed, deep-fried, salted, refined-wheat flour pockets)	46.66	13.62	29.19	49.16	41.56	8.99	0.29
*Pakoras* (deep-fried, graham flour–coated bread triangles)	54.16	16.57	30.59	46.95	40.07	11.61	0.34
*Aloo tikki* (deep- or shallow-fried patty made of potato)	45.24	12.40	27.41	60.39	33.77	4.98	0.38
*Boondi ladoo* (a ball-shaped sweet made of fried graham flour granules dipped in sugar syrup)	87.84	12.82	14.59	55.68	22.61	20.81	0.44

a The weight reflects the serving size in which the snack was sold in the urban slum markets.

b Expressed as a percentage of total fatty acids.

c Fatty acid profile not available.

**Table 3:** Fatty Acid Profile of Oils Sampled in Village and Urban Slum Settings

Type of Oil	Fatty Acids (% Total Fat)			
Saturated Fatty Acids	Monounsaturated Fatty Acids	Polyunsaturated Fatty Acids	Trans Fatty Acids
Villages				
*Vanaspati*	68.8	6.9	2.2	21.9
*Desi ghee* (homemade)	69.3	24.6	1.0	2.5
*Desi ghee* (market)	57.8	6.5	5.5	29.9
Urban slum				
Mustard oil				
A	44.1	32.2	15.6	5.2
B	24.7	40.3	32.6	0.8
C	35.1	18.1	36.6	2.9
Refined oil				
A	47.9	41.4	11.1	0.1
B	29.1	18.8	51.7	0.3
*Vanaspati*	62.8	9.5	2.4	25.3
*Desi ghee* (homemade)	69.3	24.6	1.0	2.5
*Desi ghee* (market)	58.8	2.7	34.5	3.9

Note: Three varieties of mustard oil and 2 varieties of refined oil were sampled from the urban slum community. A, B, and C distinguish the different varieties of oils.
